# AGING IN LATIN AMERICA: A FOCUS ON MIDDLE-INCOME COUNTRIES

**DOI:** 10.1093/geroni/igz038.2899

**Published:** 2019-11-08

**Authors:** Catherine Garcia, Maria P Aranda

**Affiliations:** University of Southern California, Los Angeles, California, United States

## Abstract

Population aging is occurring rapidly across Latin America, a region that includes some of the world’s most racially, ethnically, and culturally diverse populations. Aging in this region is occurring in a context of high levels of poverty and income inequality, which has implications for disease risk, cognitive health, and overall well-being. This symposium focuses on Mexico and Colombia, two of Latin America’s largest middle-income countries, which have recently undergone rapid epidemiological and demographic transitions. The papers in this symposium examine a variety of health dimensions among older Latinos that include physiological functioning, cognition, and psychological and physical well-being. García uses the Mexican Health and Aging Study (MHAS) and the Health and Retirement Study (HRS) to examine biomarkers known to predict health risk among Mexican-origin populations: Mexico-born living in Mexico, Mexico-born living in the U.S., and U.S.-born Mexican-Americans. Saenz examines the importance of education on late-life cognitive ability among Mexicans using data from the MHAS Cognitive Aging Ancillary Study. Using data from the Colombian Survey of Health, Well-Being, and Aging (SABE-Colombia), Ailshire examines variation in biological risk across key subgroups of the population. Osuna uses data from the Colombian National Quality of Life Survey (ENCV) to determine if social and economic inequalities are reflected in unequal health and well-being among older adults. Results highlight which Latin American populations have increased risk for poorer health, which merit further research and policy attention. The findings highlight the importance of understanding health and well-being in the rapidly growing older adult populations of Latin America.

